# Where Is Garlic Mustard? Understanding the Ecological Context for Invasions of *Alliaria petiolata*

**DOI:** 10.1093/biosci/biac012

**Published:** 2022-03-30

**Authors:** Vikki L Rodgers, Sara E Scanga, Mary Beth Kolozsvary, Danielle E Garneau, Jason S Kilgore, Laurel J Anderson, Kristine N Hopfensperger, Anna G Aguilera, Rebecca A Urban, Kevyn J Juneau

**Affiliations:** Babson College, Babson Park, Massachusetts, United States; Rodgers and Scanga are co-first authors of this article; Utica University, Utica, New York, United States; Rodgers and Scanga are co-first authors of this article; Siena College, Loudonville, New York, United States; State University of New York Plattsburgh, Plattsburgh, New York, United States; Washington and Jefferson College, Washington, Pennsylvania, United States; Ohio Wesleyan University, Delaware, Ohio, United States; Northern Kentucky University, Highland Heights, Kentucky, United States; Simmons University, Boston, Massachusetts, United States; Lebanon Valley College, Annville, Pennsylvania, United States; University of Wisconsin–River Falls, River Falls, Wisconsin, United States

**Keywords:** garlic mustard (Alliaria petiolata), invasive species, spatial and temporal scaling, macrosystems, ecological context

## Abstract

The invasive plant Alliaria petiolata (garlic mustard) has spread throughout forest understory and edge communities in much of North America, but its persistence, density, and impacts have varied across sites and time. Surveying the literature since 2008, we evaluated both previously proposed and new mechanisms for garlic mustard's invasion success and note how they interact and vary across ecological contexts. We analyzed how and where garlic mustard has been studied and found a lack of multisite and longitudinal studies, as well as regions that may be under- or overstudied, leading to poor representation for understanding and predicting future invasion dynamics. Inconsistencies in how sampling units are scaled and defined can also hamper our understanding of invasive species. We present new conceptual models for garlic mustard invasion from a macrosystems perspective, emphasizing the importance of synergies and feedbacks among mechanisms across spatial and temporal scales to produce variable ecological contexts.

Context dependency, which occurs as a result of complex interactions among mechanistic factors, is increasingly recognized in complex, macroscale, strongly empirical fields of study such as ecology. Invasion studies may be particularly prone to context dependency because of the wide variety of interactions encountered and initiated by the invader in its novel environment (Catford et al. 2022). Typically, invasion ecology focuses on either intrinsic species-specific characteristics or extrinsic (abiotic and biotic environmental) factors (Colautti et al. 2014), but these mechanisms interact and can jointly affect the success or failure and magnitude of invasion (Sapsford et al. 2020). Recent studies have recognized and promoted awareness of the complexity that arises from these interacting mechanisms and emphasized the importance of ecological context to the outcomes of invasions (Kumschick et al. 2015, Sapsford et al. 2020).

Alliaria petiolata (M. Bieb.) Cavara and Grande (garlic mustard, Brassicaceae) is often used as a model or case study for plant invasion because it currently has a wide distribution, is considered a primary threat to hardwood forests, has been widely studied (Barney and Whitlow 2008, Colautti et al. 2014), and has recently had its genome sequenced (Alabi et al. 2021), which makes it an ideal species to elucidate the influence of ecological context on invasion success. As a western Eurasian plant, garlic mustard was likely introduced to North America by early colonists as a medicinal plant and garlic substitute (Grieve 1959). Garlic mustard has a strict biennial life cycle with a rosette stage in the first year that overwinters green to produce a flowering stalk in the second year (Cavers et al. 1979). Garlic mustard was first formally identified in North America in the 1860s in Long Island, New York, and has since invaded a range of forest understory and edge communities across the continent (figure 1a; Nuzzo 1993). However, its persistence, density, and impacts, such as decreasing native plant diversity and growth, mycorrhizal fungi abundance, and native butterfly survival, as well as altering soil nutrient cycling (Rodgers et al. 2008a, 2008b), have widely varied across sites and changed over time (Lankau et al. 2009, Cipollini and Cipollini 2016, Haines et al. 2018). Yet similar to other invasive species, research on garlic mustard has primarily focused on single sites, single time points, and responses by single native species despite the acknowledged variability in garlic mustard success across its nonnative range and from year to year.

**Figure 1.**
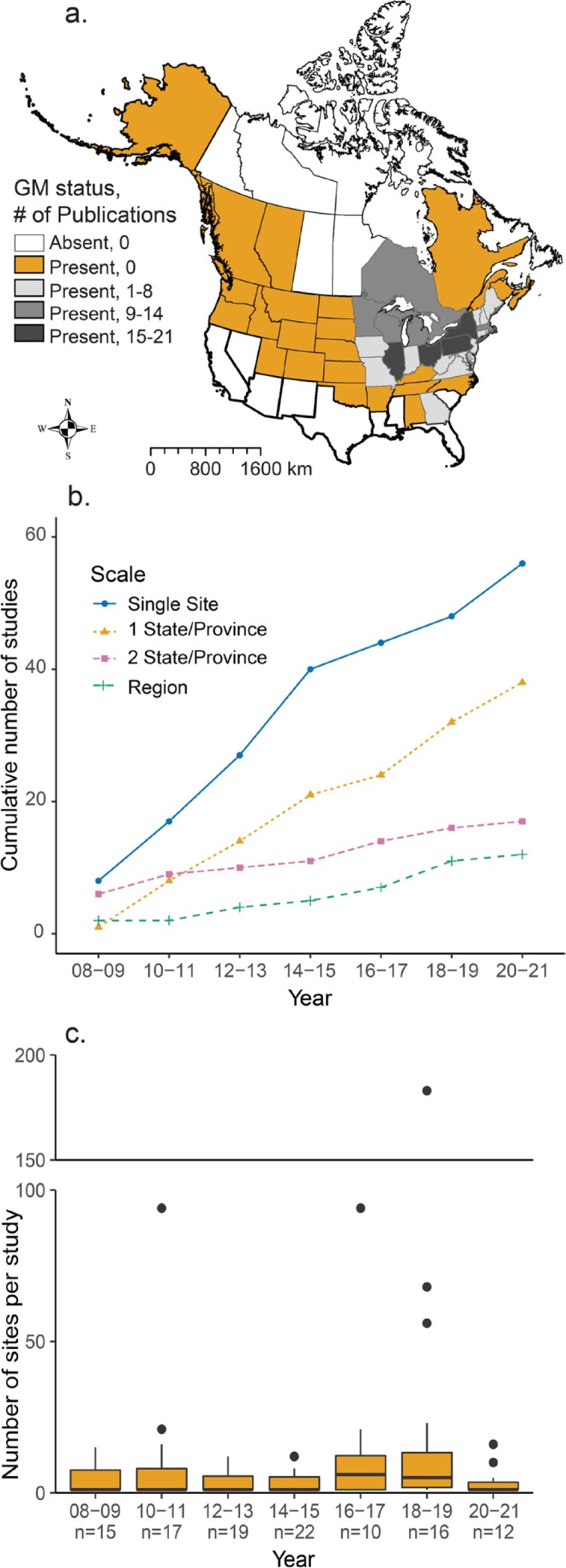
Site-based research on invasive garlic mustard at various scales in the published literature between 1 January 2008 and 31 December 2021 (represented by last two digits of year). (a) The number of publications since 2008 with at least one site in each state or province, superimposed over the range of garlic mustard (GM, states or provinces where it is present). Garlic mustard range (state-level presence) was estimated based on data from the Center for Invasive Species and Ecosystem Health at the University of Georgia's Early Detection and Distribution Mapping System (EDDMapS) and iNaturalist GBIF Research Grade observations. (b) The cumulative number of studies over time conducted at different spatial scales, including single site within one state or province or multisite within one, two, or more states or provinces. (c) The number of sites per study over time. The sample size (n) for the boxplots is indicated under the year.

Landscape-scale characteristics perform well in predicting the risk of invasion for most species, but their effects are species specific, and generalized risk-of-invasion models fail to account for site-specific conditions that may promote or deter invasion (Lázaro-Lobo et al. 2020). Thirteen years after reviewing the inevitability of garlic mustard as a permanent member of eastern North American forests (Rodgers et al. 2008a), in the present article, we survey literature published since 2008 to revisit the proposed mechanisms underlying garlic mustard invasion and its impacts and to determine their generality across ecological contexts, analyze how and where garlic mustard has been studied, highlighting examples of multisite and multiple-time-point studies, and present updated conceptual models for garlic mustard invasion success that emphasize the importance of ecological context, which is a function of environmental factors operating and interacting across spatial and temporal scales.

## Mechanisms for garlic mustard invasion success across ecological contexts

Rodgers and colleagues (2008a) proposed seven distinct mechanisms for the invasion success of garlic mustard: the release of secondary compounds, soil biota and soil chemistry feedbacks, escape from natural enemies and herbivory, competitive ability, extended growing season (although Rodgers et al. 2008a referred to this as early phenology), high phenotypic plasticity, and high reproductive output. In several cases, these mechanisms are garlic mustard-specific examples of broader hypotheses that have attempted to generalize mechanisms for invasive plant success across ecosystems, such as fluctuating resources (Davis et al. 2000), enemy release (Elton 1958, Maron and Vilà 2001), optimal defense (Coley et al. 1985, Bazzaz et al. 1987), novel weapons (Barto et al. 2010), and evolution of increased competitive ability (Blossey and Notzold 1995). However, research since 2008 has shown that many of these mechanisms for predicting the success of garlic mustard invasion are dependent on ecological context, meaning that the same level of a variable leads to different degrees of invasion success in different sites, because of interactions with other site variables. A recent review evaluated the 10 leading proposed mechanisms for invasion of understory forest herbs and found that single species often had multiple mechanisms driving invasion success and that the results for single invaders differed across space and time (Wavrek et al. 2017). Different mechanisms operate at different scales (Fridley et al. 2007), and therefore one single mechanism is unlikely to be entirely responsible for the success of any individual plant species (Liebhold et al. 2017). In addition, as with many other invasions, differentiating whether garlic mustard invades because of specific preexisting environmental site conditions or if garlic mustard is altering conditions for its own postinvasion success is difficult, begging the question as to whether garlic mustard is a passenger or a driver of change (MacDougall and Turkington 2005, Phillips-Mao et al. 2014, Anderson et al. 2019) in invaded sites. We begin in the present article by considering recent (2008–2021) progress in the literature regarding the mechanisms proposed by Rodgers and colleagues (2008a).

### Release of secondary compounds and soil biota feedbacks

Garlic mustard has a unique phytochemical profile compared with native North American mustard plants (Barto et al. 2010, Frisch et al. 2014), which suggests that the novel weapons hypothesis may play a role in garlic mustard success (Callaway and Ridenour 2007). The secondary compounds in garlic mustard have been identified and widely studied, and are known to have variable efficacy as allelopathic chemicals (Pisula and Meiners 2010, Cipollini and Bohrer 2016). In particular, the degradation of sinigrin glucosinolate results in the allelopathic allyl isothiocyanate, which accounts for nearly half of the volatile contents of fresh garlic mustard leaves (Blažvić and Mastelić 2008). Cipollini and colleagues (2012) and Cipollini and Flint (2013) found that garlic mustard leaf and shoot extracts, more than root extracts, inhibited seed germination in plants from the Asteraceae and Lamiaceae families but had less of an impact on other species in the Brassicaceae family. Whereas garlic mustard leaf extracts reduce growth in some native plants (e.g., Brouwer et al. 2015), nonmycorrhizal species are less affected (Cipollini et al. 2008). Compared with extracts from other invasive plants, garlic mustard extracts appear to be intermediate in their inhibitory effect on seed germination, plant biomass, and mycorrhizal association (Cipollini and Bohrer 2016).

The allelopathic effects of garlic mustard also vary across life stage (Evans et al. 2016), individuals (Lankau, 2010, 2011a, Frisch et al. 2014), and populations (Hillstrom and Cipollini 2011), and shift over time because of complex interactions with other plants and ecological contexts (Cipollini and Cipollini 2016, Evans et al. 2016). Garlic mustard displays defense traits that are strongly inducible and, when invoked, incur a significant cost to its own vegetative growth, suggesting an important trade-off between defense and individual plant success (Cipollini and Lieurance 2012, Harris 2018). Allelochemical production varies within a population (Lankau 2011b), likely because of physiological plasticity in response to a plant's environment, such as light resources (Smith 2015, Harris 2018). The bioactive chemistry of garlic mustard also varies by season (Haribal and Renwick 2001), population (Hillstrom and Cipollini 2011), and broader environmental site conditions (Cipollini 2002, Lankau 2010, Hillstrom and Cipollini 2011, Smith and Reynolds 2015). Cipollini and Cipollini (2016) show that the magnitude of allelopathic effects is influenced by garlic mustard population density and age, the legacy effect of garlic mustard on soil and microbial communities, and dependencies of target plants on mutualistic associations with microbes.

Although previous invasion by other species can facilitate garlic mustard success (Flory and Bauer 2014), garlic mustard allelopathic effects can be masked by the stronger effects of resource competition among native plants (Barto and Cipollini 2009a), given that allelopathic compounds are often undetectable in soil beneath garlic mustard plants (Barto and Cipollini 2009b) and that these compounds rapidly degrade in nonsterile soils (Gimsing et al., 2006, 2007, Barto and Cipollini 2009b). Furthermore, the allelopathic effects of garlic mustard are predicted to decline in the future because phytotoxin production decreases in older populations (Lankau et al. 2009, Huang et al. 2018) and under higher atmospheric carbon dioxide concentrations and warmer spring temperatures (Anderson and Cipollini 2013), and native plants develop competitive tolerance (Lankau 2012a).

Allelochemicals produced by garlic mustard often indirectly affect the plant community via the soil biota, but these demonstrated impacts are also complicated by ecological context. Some studies showed that garlic mustard decreases soil microbial community richness (Lankau 2011b) and fungal hyphae networks (Poon and Maherali 2015, Hale et al. 2016) and reduces success of arbuscular mycorrhizal fungi, ectomycorrhizal fungi, and entomopathogenic fungi (Roberts and Anderson 2001, Wolfe 2002, Stinson et al. 2006, Callaway et al. 2008, Keesing et al. 2011, Vaicekonyte and Keesing 2012, Portales-Reyes et al. 2015). However, no studies were definitive, because of confounding variables. Other studies either showed no impact of garlic mustard on soil biota or a weak effect with other stronger ecosystem drivers (Burke 2008, Barto et al. 2010, Koch et al. 2011, Phillips-Mao 2012). Confounding variables influencing the impact of garlic mustard on the soil biota include the presence of white-tailed deer (Odocoileus virginianus; Burke et al. 2019), concentration of soil nutrients, particularly nitrogen (Castellano and Gorchov 2012, Poon and Maherali 2015, Anthony et al. 2020, Cope et al. 2021), and time since invasion (Barto et al., 2010, 2011, Lankau, 2010, 2011b, Davis et al., 2012, 2014, 2015, Blossey et al. 2021).

Ecosystem recovery after garlic mustard removal is complex with current findings showing that ectomycorrhizal fungi abundance increases in the short term (Vaicekonyte and Keesing 2012), but overall mycorrhizal community recovery is slow with impacts from garlic mustard persisting in the long term (Anderson et al. 2010, Lankau et al. 2014, Burke et al. 2019, Roche et al. 2021). In addition, Lankau (2012a) found that native plants that co-occur with garlic mustard were better at maintaining arbuscular mycorrhizal fungi root colonization when in the presence of the invader than native plants that came from uninvaded sites. Recent work by Duchesneau and colleagues (2021) suggests that garlic mustard is a driver of change through proliferation of soil pathogens and changes to nitrogen-cycling microbial groups. In summary, the allelopathic influences of garlic mustard on soil biota are one of this species’ most widely known and most intensively studied effects on invaded ecosystems, but also show some of the most variability across different studies and ecological contexts.

### Native plant competition

Although invasive plants are often assumed to be better competitors than native species (Baker 1965), garlic mustard is not an overwhelming competitor with other understory plant species when grown head to head (Meekins and McCarthy 1999, Wixted and McGraw 2010, Davis et al., 2012, 2015, Leicht-Young et al. 2012, Anderson et al. 2019, Faison et al. 2019). This holds true even when competing with a sympatric, co-occurring invasive mustard species with a similar life history strategy and growth form (dame's rocket, Hesperis matronalis; Leicht-Young et al. 2012). Although garlic mustard presence can reduce native plant cover (Burke et al. 2019), high coverage from native plants also suppresses garlic mustard establishment and growth (Phillips-Mao et al. 2014). Furthermore, pathogens like powdery mildew (Erysiphe cruciferarum) may reduce the competitive impact of garlic mustard in the future (Cipollini and Enright 2009). However, there are ecological contexts in which garlic mustard coexists more often with local native plants rather than with other invasives (Gavier-Pizarro et al. 2010, O'Sullivan et al. 2019), in which native species exhibit genetic variability in their competitive interactions with garlic mustard (Gibson et al. 2014), and in which native plants have evolved resistance to garlic mustard (Cipollini and Hurley 2008, Huang et al. 2018). Even where garlic mustard presence reduces the growth of some native plants, it can increase the growth and survival of others (Waller and Maas 2013).

Garlic mustard's negative impact on some native herbs and trees with mycorrhizal associations has been found to be due to reduced fungal association causing reduced water absorption, leading to carbon stress for the native plants (Hale et al. 2016). However, soil legacy effects of garlic mustard presence may take decades to play out in the form of changes in native plant communities (Dornbush and Hahn 2013). Interestingly, second-year garlic mustard plants are also an important competitor with the first-year rosettes; intraspecific competition is likely the cause of alternating dominance between the two cohorts (Bauer et al. 2010, Davis et al. 2012), creating a strong biennial pattern at most sites (Pardini et al. 2009). However, extreme climate events can disrupt this alternating pattern by reducing the abundance of garlic mustard rosettes (Anderson et al. 2021). Overall, studies of garlic mustard interactions with native plants show that garlic mustard is not gaining an advantage over native species through direct, strong competition for resources.

### Garlic mustard–animal interactions

Direct and indirect interactions of white-tailed deer, nonnative earthworms, butterflies, and spiders with garlic mustard add to the complexity of the invasion story. Garlic mustard is eaten by a diverse community of herbivores in its native Europe but lacks specialist herbivores in North America (Blossey et al. 2001), indicating that the enemy release hypothesis (Elton 1958, Wolfe 2002) plays a strong role in garlic mustard's invasion success. Although some invertebrates have been observed feeding on garlic mustard in North America (Yates and Murphy 2008), garlic mustard typically suffers minimal herbivory damage in the field (Lewis et al. 2006, Van Riper et al. 2010, Hahn et al. 2011, Averill et al. 2018) and in feeding trials (Averill et al. 2016), although there is some evidence that herbivory impacts vary across sites. For example, Biswas and colleagues (2015) found higher rates of herbivory in grasslands than forests, and in second-year plants than in first-year rosettes. Overall, however, generalist herbivores and pathogens appear to do little to curtail garlic mustard growth and spread in North America (Blossey et al. 2001).

At the regional scale, increasing deer density can reduce native and overall plant community richness, diversity, and abundance (Averill et al. 2018). In most cases, garlic mustard benefits from abundant white-tailed deer, which preferentially feed on native understory flora, removing competitors for resources and therefore supporting higher densities of the invader (Knight et al. 2009). In fact, Kalisz and colleagues (2014) demonstrated that deer were required for garlic mustard success in some sites. Reducing or eliminating deer browse pressure in forested sites results in a decline of nonnative vegetation (including garlic mustard) cover, abundance, growth, and population growth rates (Kalisz et al. 2014, Dávalos et al. 2015a, Blossey et al. 2017a), suggesting that herbivore-mediated plant selection promotes garlic mustard expansion. Although interesting exceptions to the positive association between deer and garlic mustard success have been observed in certain ecological contexts, such as in the heavily grazed suburban forests studied by Morrison and colleagues (2021), previous work has been consistent in showing a positive relationship between deer herbivory on native plants and garlic mustard success.

Earthworms, as ecosystem engineers, have broad-reaching effects on ecosystems (Le Bayon et al. 2017), but their role in facilitating the expansion of garlic mustard is unclear. In general, nonnative earthworm biomass is positively associated with invasive plant cover (including garlic mustard) and negatively associated with native plant presence (Nuzzo et al., 2009, 2015, Craven et al. 2017), and removal of garlic mustard decreases nonnative earthworm biomass (Stinson et al. 2018). However, Hopfensperger and Hamilton (2015) found a negative relationship between garlic mustard cover and the proportion of immature nonnative earthworms, which they speculated may be associated with allelopathy. Anecic (vertical burrowing) earthworm species reduce garlic mustard success because of their distribution and movement through the soil layers. For example, anecic common nightcrawlers (Lumbricus terrestris) transport, disperse, and digest garlic mustard seeds (Nuzzo et al. 2009), preferring garlic mustard in seed-choice experiments (Quackenbush et al. 2012, Cassin and Kotanen 2016) and exhibiting density-dependent consumption (more than a 300% increase in consumption with an experimental doubling of seed density; McTavish and Murphy 2019). Furthermore, among 23 plant species tested, smaller seeds (including garlic mustard) were consumed and digested more readily and relocated to greater soil depths, reducing their likelihood of germination and further complicating the role of nonnative earthworms in the invasion process at the landscape scale (Cassin and Kotanen 2016).

Deer and earthworms synergistically interact in a complex manner, by deer selectively browsing on native plants, whereas earthworms alter soil density and nutrient levels, reduce mycorrhizal associations, and consume plant root hairs (Dávalos et al. 2015b, 2015a, 2015c). In addition, Karberg and Lilleskov (2009) showed that the common nightcrawler benefits from the addition of nutrient-rich deer fecal pellets in forests. Deer and earthworm densities vary across sites, and the strength of their interactions and the resulting influence on garlic mustard invasion likely varies across spatial and temporal scales. A long-term comparative multisite and multistate study showed that although deer presence influences nonnative earthworm abundance, deer herbivory is the ultimate driver of native species loss (Nuzzo et al. 2017).

Additional garlic mustard–animal interactions and their effects (e.g., animal-mediated seed dispersal, evolutionary traps, understory structural complexity) further illustrate how ecological context can influence invasion dynamics. For example, seed dispersal experiments with white-tailed deer and raccoon (Procyon lotor) pelts provide evidence for the potential of garlic mustard long-distance seed dispersal, with implications for greater garlic mustard movement across landscapes (Loebach and Anderson 2018). The use of garlic mustard stems and seeds has been documented in nest construction by veeries (Catharus fuscescens; Heckscher et al. 2014), although the potential contribution to garlic mustard seed dispersal is limited, because veeries typically collect material for nest construction close to the nest site (Heckscher 2007). Furthermore, garlic mustard's chemical composition and height advantage may lead native butterfly species to select the invader over native species for oviposition (Davis and Cipollini 2014). As such, garlic mustard may serve as an evolutionary trap for native butterflies, leading to markedly reduced larval butterfly survival, growth, and delayed pupation (Keeler and Chew 2008, Davis and Cipollini 2014, Davis et al. 2015, Morton et al. 2015, Augustine and Kingsolver 2018), especially in stressful environments (Bauerfeind and Fischer 2013). Garlic mustard patches are also associated with higher spider densities than nongarlic mustard patches (Smith and Schmitz 2015, Smith-Ramesh 2017), and senesced garlic mustard siliques increase structural complexity of the forest understory (Smith and Schmitz 2015, Smith-Ramesh, 2017, 2018, Landsman et al. 2021), leading to trophic restructuring that shifts feeding behavior toward aerial insects, and ultimately results in reduced survival of sit-and-wait predators, especially wolf spiders (Lycosidae; deHart and Strand 2012).

Collectively, studies on the interactions of garlic mustard with animals indicate that deer facilitate the success of garlic mustard at the expense of native plant communities, and this is primarily accomplished through their preferential feeding activities and movements. In addition, garlic mustard, through its chemical composition or contribution to the structural complexity of understory vegetation, leads to trophic restructuring of insect and arachnid communities and serves as an evolutionary trap for native butterflies, especially in stressful environments. The role of nonnative earthworms and other species (e.g., veeries, raccoons) in garlic mustard success is more variable across studies and has not been investigated extensively.

### Extended growing season and light availability

Extended growing season, sometimes referred to as extended leaf phenology, has emerged as a mechanism for the successful invasion of a number of nonnative plants in the forest understory (e.g., Wolkovich and Cleland 2011, Smith 2013), including garlic mustard (Engelhardt and Anderson 2011, Smith and Reynolds 2015, Heberling et al. 2019). Initially, garlic mustard's success was attributed to its early spring emergence giving it access to light availability prior to canopy closure (Lapointe 2001), but now, its extended growing season into the autumn months, including its unique access to autumn irradiance by rosettes overwintering green (Heberling et al. 2019), is also recognized as a potential mechanism for garlic mustard success (Smith and Reynolds, 2014, 2015). Nutrient pulses in the fall increase garlic mustard biomass, but pulses in the spring reduce biomass (Heckman and Carr 2015), suggesting that garlic mustard can take advantage of additional nutrients in the fall while capitalizing on extended light resources in the spring. The advantage of extended growing season can be expected to keep pace with warming temperatures because of climate change; garlic mustard seedling emergence and growth has been shown to positively correlate with spring temperatures from populations in the eastern United States and the United Kingdom, as well as experimental warming treatments (Anderson and Cipollini 2013, Blossey et al. 2017b, Footitt et al. 2018, Fox and Jönsson 2019).

Garlic mustard is also able to exploit increased light availability due to ephemeral canopy disturbances (Eschtruth and Battles 2009). High light conditions increase garlic mustard photosynthesis (Myers and Anderson 2003, Myers et al. 2005, Engelhardt and Anderson 2011), survival (Smith and Reynolds 2015), and seed production (Engelhardt and Anderson 2011, Stinson and Seidler 2014). Canopy disturbance, propagule pressure, and their interaction were found to be more important predictors of garlic mustard invasion than species diversity or herbivory (Eschtruth and Battles, 2009, 2011, 2014, Biswas and Wagner 2015). Canopy disturbance also directly influences propagule pressure, because increased light availability leads to increased seed production (Meekins and McCarthy 2000, Phillips-Mao 2012, Stinson and Seidler 2014, Huebner et al. 2018). For example, Eschtruth and Battles (2014) showed that canopy disturbance created by dispar moth (Lymantria dispar dispar) defoliation increases local seed production and propagule pressure, resulting in increased abundance of garlic mustard. However, there are exceptions. Some studies have reported that garlic mustard performance is not positively correlated with light availability (Kunkel and Chen 2021) and that garlic mustard biomass increases with canopy coverage (Gavier-Pizarro et al. 2010, Smith and Reynolds 2015, Warren et al. 2015). Ultimately, it seems that high light availability is likely to be important to garlic mustard success, although it may not be the most important driver in all contexts, and garlic mustard's extended growing season allows it to take advantage of seasonal and environmental variations in light and nutrients.

### Reproductive output, seed dispersal, and seed bank

Reproductive output for garlic mustard is site-specific with seed densities ranging by an order of magnitude from 9500 seeds per square meter in northern Illinois (Nuzzo 1993) to 107,000 seeds per square meter in Ontario (Cavers et al. 1979). Seed abundance per individual and per silique is positively correlated with plant height, regardless of conspecific density (Smith et al. 2003), and reproductive effort represents 20% of a plant's total biomass (Anderson et al. 1996), suggesting that environmental factors that affect plant growth also affect propagule volume. High reproductive output facilitates garlic mustard spread into gaps or open habitats en route to understory forest microhabitats, where its growth and survival traits, such as extended growing season, are favored (Meekins and McCarthy 2000, Kunkel and Chen 2021). Edge-interior microsites display source–sink dynamics (Stinson and Seidler 2014), because populations in edge habitats produce significantly more seeds than those in intermediate and interior sites (Stinson et al. 2019). Although seed dispersal distance has been assumed to be within a few meters (Nuzzo 1999) in models exploring propagule pressure (Eschtruth and Battles 2009), empirically measured distances range from 0.52 (Loebach and Anderson 2018) to 1.82 (Biswas and Wagner 2015) meters. Given this low dispersal distance, garlic mustard would remain relatively localized without long-distance dispersal, such as that facilitated by humans or other animals (see the “Garlic ­mustard–animal interactions” section). In fact, the rate of spread has been found to increase; two centuries of herbarium data reveal that after the establishment phase, when garlic mustard spread beyond the northeastern United States, the rate of spread tripled in the 1960s and this faster spread has held constant (Clark et al. 2018).

Persistent seed banks allow locally adapted seeds to wait for the appropriate cues to germinate, which facilitates the leading edge of an invasion to successfully encroach into new ranges or niches with shifting climates, thereby facilitating new invasions (Blossey et al. 2017b, Redwood et al. 2018, Presotto et al. 2020). Redwood and colleagues (2018) show that up to 88% of garlic mustard seeds survive in the seedbank after 2 years, and Blossey and colleagues (2017b) found that seeds can remain viable for 13 years or more in the seed bank. Furthermore, just as seed production varies with site characteristics, such as light availability (see the “Extended growing season and light availability” section), the behavior of the seed bank is influenced by ecological context (Blossey et al. 2017b, Yasin and Andreasen 2018). For example, garlic mustard shows increased germination rates in hypoxic conditions created by leaf litter (Yasin and Andreasen 2018). Clearly, the size of the seed bank is also influenced by the other factors of reproductive output and seed dispersal discussed in the present article—that is, fecundity and propagule pressure. Whereas high reproductive output, facilitated seed dispersal, and persistent seed banks must be positive contributors to garlic mustard success, site-specific factors can modify these drivers, leading invasion success to change over space and time.

### Evolutionary processes

Garlic mustard's invasion history has contributed to high overall genetic diversity in its introduced range (Meekins et al. 2001, Durka et al. 2005, Rodgers et al. 2008a), suggesting high evolutionary potential (e.g., Lavergne and Molofsky 2007). Population-level genetic diversity is lower within than among populations, implying multiple introductions (Meekins et al. 2001, Durka et al. 2005, Cipollini et al. 2020) followed by range expansion (Clark et al. 2018) to form new populations that undergo local genetic drift (Mullarkey et al. 2013) or possibly natural selection leading to local adaptation.

Evidence for local adaptation in garlic mustard's introduced range is mixed and depends in part on the spatial scale at which particular traits have been examined. At subregional scales, little evidence exists for local adaptation; for example, no local adaptation was detected in glucosinolate production in response to light (Smith 2015) or in fitness-related traits across microhabitats (e.g., edge versus forest interior; Stinson and Seidler 2014, Stinson et al. 2019). However, studies at larger, regional spatial scales have shown rapid local adaptation of garlic mustard in seed germination, seedling emergence in response to climate parameters (Blossey et al. 2017b), and root glucosinolate concentration in response to intra- and interspecific competition (Lankau et al. 2009, Lankau 2012a, Evans et al. 2016, Huang et al. 2018). Rapid evolution toward lower glucosinolate production may mitigate garlic mustard's invasion success and impacts, suggesting that garlic mustard may be approaching its “evolutionary limits” (Lankau et al. 2009), at least with regards to secondary compound production.

Similar to some other invasive plant species (Baker 1965), garlic mustard has high phenotypic plasticity across a variety of traits in response to environmental variation (e.g., Byers and Quinn 1998), which has been found to contribute to its invasion success (Richards et al. 2006). For example, garlic mustard shows phenotypic plasticity in growth, reproduction, photosynthetic activity (Stinson and Seidler 2014), and glucosinolate production in response to variation in light availability (Smith 2015). Chemical defenses and leaf traits also exhibit plasticity in response to water and nutrient availability and exposure to jasmonic acid (Hillstrom and Cipollini 2011). Although the degree of expression of plasticity seems to vary by population, suggesting plasticity traits have the potential for local adaptation (e.g., Hillstrom and Cipollini 2011), the extent to which phenotypic plasticity confers fitness benefits to garlic mustard remains unclear (Stinson et al. 2019). No distinct patterns of phenotypic plasticity emerge across the continents for garlic mustard's native and introduced ranges (Hillstrom and Cipollini 2011, Cipollini et al. 2020), but retention of that plasticity may be more advantageous in its introduced range. For example, expressing induced rather than constitutive defenses may allow garlic mustard to allocate resources to growth in the introduced range, where natural enemies are scarce or lacking (Cipollini and Lieurance 2012), thereby providing competitive advantages (Cipollini et al. 2020).

Garlic mustard has also triggered evolutionary changes in other organisms, including native plants (e.g., Cipollini and Hurley 2008, Lankau 2012a, 2013, Lankau and Nodurft 2013, Huang et al. 2018), soil microbes (Lankau 2011b), and butterflies (Morton et al. 2015). Research thus far has not yet disentangled the complicated interactions among phenotypic plasticity, local adaptation, and possible preadaptation (Blossey et al. 2017b) on garlic mustard invasion success. In addition, factors such as maternal effects (e.g., Stinson and Seidler 2014, Blossey et al. 2017b, Stinson et al. 2019) and changing climate conditions (Anderson and Cipollini 2013, Footitt et al. 2018) will continue to complicate these efforts. Coevolution between garlic mustard and the native species in its introduced range (e.g., Lankau 2012a, Huang et al. 2018) will also produce context-dependent feedbacks that change garlic mustard's invasion success and impacts over both space and time.

### Soil characteristics

Garlic mustard's relationship with soil nutrients is complex. Although garlic mustard is known to be effective at nutrient uptake (Poon and Maherali 2015) and to be a nitrogen generalist (Hewins and Hyatt 2010), relationships with soil nitrogen in the field are variable. Rodgers and colleagues, 2008a, 2008b) described a positive association between garlic mustard and the availability of nitrogen and phosphorus. However, Lankau (2012a) found no significant correlations between garlic mustard cover and soil nitrate and ammonium when examining six sites across the midwestern and northeastern United States. Some work has was shown no association between garlic mustard presence and soil ammonium availability (Castellano and Gorchov 2012, Burke et al. 2019), whereas positive relationships between garlic mustard presence and nitrogen mineralization rates, total soil nitrogen (Morris et al. 2012), and soil nitrate (Castellano and Gorchov 2012, Phillips-Mao et al. 2014) have been detected in other work, although these studies were predominantly short term.

Garlic mustard lacks the mycorrhizal partners that facilitate soil phosphorus uptake for most other plants, and garlic mustard presence has been found to be positively associated with soil phosphorus availability (Castellano and Gorchov 2012, Phillips-Mao et al. 2014, Anderson et al. 2019). But work by Lankau (2012b) found no correlation between soil phosphorus and garlic mustard cover. It is unclear whether garlic mustard causes higher phosphorus levels because of increased mineral weathering (Rodgers et al. 2008b) or simply responds positively to higher phosphorus levels (Castellano and Gorchov 2012, Anderson et al. 2019). However, the removal of garlic mustard over 8 years at a site in Pennsylvania did not change soil phosphorus levels (Burke et al. 2019), suggesting that garlic mustard was responding to, and not altering, soil conditions.

Although garlic mustard grows at a wide range of soil pH levels both within (Phillips-Mao et al. 2014) and across sites (Haines et al. 2018), it is often associated with less acidic soils (Castellano and Gorchov 2012, Morris et al. 2012, Haines et al. 2018). Garlic mustard has been found to increase soil pH (Stinson et al. 2018), which may be driving higher rates of nitrogen mineralization in invaded sites (Morris et al. 2012), although some studies have also observed no effect of garlic mustard on soil pH (Burke et al. 2019). Alerding and Hunter (2013) observed a positive correlation between garlic mustard presence and springtail (detritivore) abundance, and they suggested that increased soil pH in invaded sites may be the mechanism underlying this pattern. However, Landsman and colleagues (2021) found a reduction of springtails with garlic mustard abundance and attributed this to potentially accelerated decomposition in litter-free ground cover. How garlic mustard might alkalize soils is unknown, although Alerding and Hunter (2013) hypothesized that high nitrate uptake by garlic mustard (Hewins and Hyatt 2010) could lead to increased proton uptake by the roots, as was noted by Ehrenfeld and colleagues (2001) for other invasive plants.

Other soil characteristics are inconsistent in their associations with garlic mustard across studies. Anderson and colleagues (2019) showed weak negative associations between soil carbon and garlic mustard, and Burke and colleagues (2019) documented increased carbon after garlic mustard removal, but Morris and colleagues (2012) found no differences in soil carbon between invaded and uninvaded sites except in forests that had nitrogen fixers. Anderson and colleagues (2019) showed that calcium was positively associated, whereas magnesium and potassium were negatively associated, with garlic mustard success, but Lankau (2012a) found no correlations between these nutrients and garlic mustard cover. Soil micronutrients (e.g., copper, iron, manganese, zinc, and sodium) have not been associated with garlic mustard success, although they have been measured in few studies (Lankau 2012a, Anderson et al. 2019). Garlic mustard was positively associated with higher soil moisture at regional scales (Haines et al. 2018), but other studies showed no effect of garlic mustard on soil moisture up to 8 years after removal (Stinson et al. 2018, Burke et al. 2019). In summary, the complexity and variability of findings for garlic mustard interactions with soil suggest a need to investigate the scales at which garlic mustard associates with elevated nutrient levels and other soil characteristics. In addition, more research is needed to explore whether there are interacting environmental drivers that determine how strongly garlic mustard affects soil properties across different sites.

### Current understanding of garlic mustard invasion mechanisms

Our review of the published research over the last 13 years indicates a shift in focus from identifying the inherent traits that make garlic mustard a successful invader to how site characteristics, whether prior to or after invasion, facilitate garlic mustard success (Lankau et al. 2009, Colautti et al. 2014, Stinson and Seidler 2014, Haines et al. 2018). Some mechanisms, such as strong competition by garlic mustard, phenotypic plasticity, and soil chemistry feedbacks, are likely less important as primary drivers for garlic mustard invasion success than initially expected. However, other synergistic mechanisms, such as soil biota feedbacks, deer and earthworm interactions, extended growing season coupled with high reproductive output, and coevolution with native species may be important drivers in certain ecological contexts and at certain spatial and temporal scales. A plausible scenario suggested by our review is that garlic mustard will be successful in sites where deer and earthworms reduce competition by native plants, allowing garlic mustard to gain a foothold. The invader can then intensify the poor performance of native mycorrhizal plant species through allelopathic disruption of root mutualisms and spread quickly through extended growing season and high seed set, particularly if light levels are high. With the potential for more rapid canopy closure with earlier arrival of spring, garlic mustard may experience a tradeoff between increasing survival at lower light levels and increasing seed set at higher light levels (Merow et al. 2017). Interestingly, this may further complicate garlic mustard's population growth and intraspecific competition dynamics as lower light availability may increase survival of plants in the first-year rosette stage, but decrease reproductive output in the second year adult stage. In addition, synergies among spiders, other invertebrates, and soil chemistry can affect the relative dominance of garlic mustard over native plants at some sites (Smith-Ramesh 2018, Landsman et al. 2021).

The complicated interactions and feedbacks among the inherent traits of garlic mustard, conditions in the novel environment, and other nonnative organisms can also lead to elevated success and exacerbated impacts at larger spatial and longer time scales. Lundgren and colleagues (2004) found that prior land use, in particular New England's agricultural legacy, helps to explain garlic mustard invasion success; however, this same association was not observed in Ohio (Burls and McClaugherty 2008). Potential explanations for these conflicting findings are differences in soil types and phenotypic plasticity of flowering times across environmental gradients, as well as landscape features that promote dispersal (e.g., stone walls, hedgerows, river corridors) that have been known to offset land-use legacy effects (Byers and Quinn 1998). In addition, agricultural legacy, paired with forest pests, have worked in tandem to allow for the increase in frequency and cover of garlic mustard (Katz et al. 2010). Climate change is likely further complicating interactions as warming and elevated carbon dioxide levels have been found to lower garlic mustard's allelopathic impact (Anderson and Cipollini 2013).

## How and where has garlic mustard been studied?

We suspected that identification of broad ecological patterns has been made more challenging by limitations in how and where garlic mustard has been studied. To examine this question, we used Web of Science and Google Scholar to search for all peer-reviewed primary research involving invasive garlic mustard ecology published between 1 January 2008 and 31 December 2021—that is, since the publication of Rodgers and colleagues (2008a)—using the search terms “garlic mustard” or “Alliaria petiolata” (for the Web of Science, we also included the term “invasive”). We also included any additional sources that were discovered to have cited or to be cited within these studies. We included any primary source that had our search terms in the title, abstract, or keywords, or that involved the invasion ecology of garlic mustard. Studies of garlic mustard in its native range were excluded.

We evaluated the studies to determine whether they occurred in an artificial setting (e.g., laboratory, garden) or natural field setting, and we focused our analysis on studies with a natural field component. Studies that collected their own field data for modeling or samples in the field for later analysis in the lab, including herbarium specimens, were considered natural field studies. We classified the natural field studies by the spatial scale and the number of sites sampled using the following categories: single site, multiple sites within one state or province, multiple sites within two states or provinces, or regional (i.e., incorporating three or more states or provinces). We recognize the weakness of using geographic boundaries as a proxy for ecological scale, but without consistent spatial scale information readily reported by authors across studies, this method seemed the least prone to inaccuracies. In practice, invasive plant management and policy actions often occur within geographic boundaries such as state or province borders. In addition, ecological context is likely to be more similar within a state or province or between adjacent states or provinces than among three or more states or provinces within a region.

A site was defined as a single study area that was typically contiguous with distinct ecological characteristics (i.e., ecological context) described in the publication's methods section. We would have preferred to define a site using quantitative parameters, but it was not consistently possible to determine sites on the basis of parameters such as land area or distance from other sampled units because this information was often not provided by authors. We were not able to determine the number of sites for studies that used point data because points may have occurred within the same site or within different sites.

We found 179 studies published over the 13-year period, 125 of which had a natural field component (see the supplemental material for the data set). We were able to determine the spatial scale and number of sites sampled for 123 and 111 of these field studies, respectively. About half (n = 56) of the 123 studies worked within a single site. Although appropriate for certain research questions (e.g., studying the effect of garlic mustard on an endemic species), single site studies may be prone to selection bias, resulting in an overemphasis in the literature on nonrandom case studies of high-density invasions (Rooney and Rogers 2011). The multisite studies tended to occur within only one or two states or provinces (n = 38 and n = 17, respectively), and most of the two-state or province multisite studies (n = 15 of 17) occurred in adjacent states, indicating a relatively small spatial scale. Only 12 of the 119 studies could be categorized as studying a region. Although regional studies for garlic mustard, and other invasive species (Kueffer et al. 2013), are becoming more common over time, they lag well behind single-site studies and studies occurring at multiple sites within one or two states or provinces (figure 1b).

Garlic mustard can be found across most of North America, but studies between 2008 and 2021 tended to be focused on a limited portion of its invasive range (figure 1a). Many studies published since 2008 have sampled at least one site in the northeast and midwest states and Ontario, but other regions of the range are poorly studied. Furthermore, certain sites within the well-sampled portion of garlic mustard's range have appeared in multiple studies (e.g., Trillium Trail in Pennsylvania). Returning to the same sites over time can produce important temporal data that are needed for garlic mustard, but if these studies are not integrated and a temporal analysis is not performed, then a single site's ecological context can become disproportionately represented in the literature. Expanding the scale of field studies into all parts of garlic mustard's introduced range is essential to fully understand its invasion success. This expansion is particularly essential because studies performed in different regions have shown variable results, suggesting important ecological context differences across the range of garlic mustard. For example, predictions for garlic mustard distribution in Ohio differed greatly from those for New England, where historical land use was a larger factor (Burls and McClaugherty 2008). Likewise, landscape-scale modeling based on 183 sites in Massachusetts suggested that aspects of ecological context (e.g., elevation, historical and contemporary land use) underlying garlic mustard invasion vary by region (Urbanowicz et al. 2019). Also, climate change is predicted to amplify garlic mustard invasion success in Minnesota (Reinhardt et al. 2020) but diminish its success in southern New England (Merow et al. 2017).

There was a slight trend toward sampling more sites per study in 2016–2017 (median = 6) and in 2018–2019 (median = 5), but the median number of sites per study remained close to 1 from 2008 to 2021 overall (figure 1c), and the majority of the 111 studies (n = 76, 68%) sampled fewer than five sites. In only 24 of the 111 studies were at least 10 sites sampled. Studies conducted within one or two states or provinces used a median of 6 sites (mean [M] = 19.9, standard deviation [SD] = 39.5) and 10 sites (M = 9.8, SD = 4.7), respectively, indicating an effort to increase sampling intensity and replication but not at a regional scale. Similarly, four of the five studies in which more than 50 sites were sampled from 2008 to 2021 were conducted within one state. Regional studies sampled a median of 16 sites (M = 18.6, SD = 18.3). Although results are not yet reported, the Global Garlic Mustard Field Survey (Colautti et al. 2014), which was conducted at 383 globally distributed sites, is a notable exception to the generally local nature of garlic mustard studies.

Although there is an encouraging trend toward multisite studies over the last 13 years, many of these studies are not occurring across enough sites and regions. Furthermore, when studies do occur at a larger scale they tend to be short term. Only 2 of the 12 regional (more than three state) studies since 2008 incorporated data collected over a decadal time scale (Averill et al. 2018, Blossey et al. 2021), with 2 others using herbarium specimens to estimate invasion processes over regional scales over up to two centuries (Lankau et al. 2009, Clark et al. 2018). Bialic-Murphy and colleagues (2020) used 6 years of detailed demographic data at a single site to find that garlic mustard's impact differed greatly over time and other studies have found dramatic decreases (Lankau 2012b, Murphy and McCarthy 2014, Nuzzo et al. 2017, Faison et al. 2019, Anderson et al. 2021, Blossey et al. 2021) or increases (Rooney and Rogers 2011) in garlic mustard abundance over years to decades. These findings indicate that annual and short-term studies are poor representatives of future dynamics and point to the strong need for longitudinal studies of garlic mustard invasion (Lankau et al. 2009, Blossey et al. 2021). Longitudinal studies will also help to detect effects of climate change, which is likely to produce long-term changes to garlic mustard invasion processes (Merow et al. 2017, Reinhardt et al. 2020).

## Proposing conceptual models for garlic mustard invasion success

**Figure 2.**
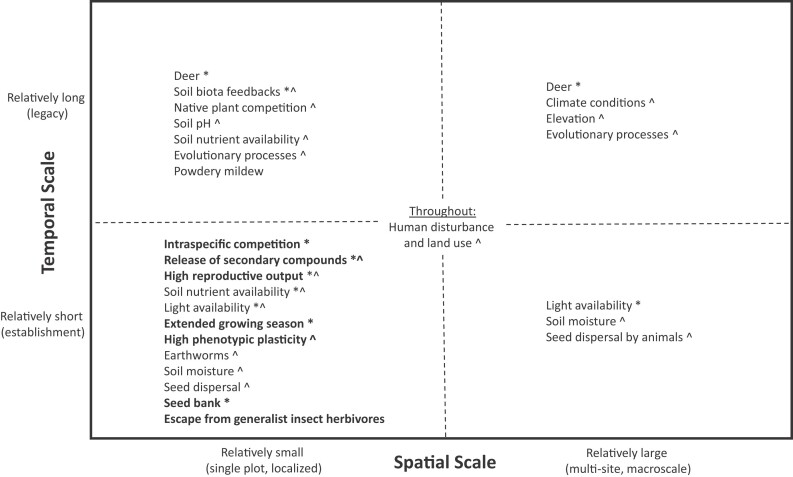
Proposed mechanisms for garlic mustard success based on the current literature, including both inherent traits of the plant (indicated in bold) and ecological context, across four categories of relatively small or large spatial scales and relatively short or long temporal scales. Mechanisms that have been shown to be most important and prevalent across different contexts are identified by an asterisk (*). Mechanisms that have been shown to be most variable across different contexts are identified by a caret (^).

Given the synergies among garlic mustard traits and various ecological contexts (Smith-Ramesh 2018), potential mechanisms for garlic mustard invasion are best considered together and with acknowledgment of their respective spatial and temporal scales and potential interactions (figure 2). Influences at smaller spatial scales can accumulate to have larger-scale site- or landscape-level influence over time (i.e., cross-scale emergences), but the conceptual model shown in figure 2 illustrates how the inherent traits of garlic mustard and variability in ecological context combine and potentially interact to provide advantages and disadvantages to garlic mustard at different scales.

Most studies have been performed on relatively small areas over short periods of time, and the multiple mechanisms that may be driving these localized populations can be seen in the bottom left area of figure 2. However, these localized populations are experiencing the effects of factors operating at both small and large spatial and temporal scales. For example, even within single plots, soil nutrient availability may interact with garlic mustard success differently at different phases of invasion, including the short-term (establishment), midterm (competition for nutrients and gradual changes in soil chemistry), and long term (legacy; figure 2). Invasion of larger spatial scales over long periods of time may be predominantly driven by deer (Heberling et al. 2017, Nuzzo et al. 2017, Burke et al. 2019), regional climate conditions (Anderson and Cipollini 2013, Merow et al. 2017, Anderson et al. 2021), land-use legacy (Lundgren et al. 2004, Katz et al. 2010), and human disturbance (Kunkel and Chen 2021). However, population-level processes such as intraspecific competition or evolutionary changes may limit long-term invasion success. These complicated synergies among factors may be site specific, thus explaining why garlic mustard is invasive in some locations but not in other ostensibly similar environments.

**Figure 3.**
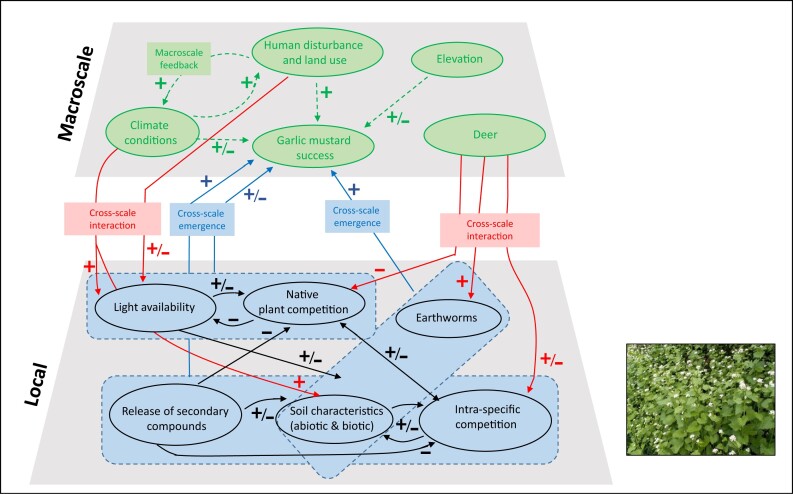
Hierarchical macrosystem model following Heffernan and colleagues (2014), identifying key variables and synergies within and across the local and macroscales for garlic mustard success. Variables are indicated as having primarily positive and/or negative influence on the connecting variable. Dispersal (not shown) causes teleconnections and alters synergies at multiple scales (see the text for details). The black arrows indicate local scale interactions, red arrows indicate cross-scale interactions, blue arrows indicate cross-scale emergences, and green dashed arrows indicate macroscale interactions. The dashed blue boxes indicate the local variables that interact to produce cross-scale emergences.

The interactions of the mechanisms in figure 2 and evidence from the post-2008 literature highlight the need for further exploration of garlic mustard invasion using a macrosystems approach. Macrosystems ecology explores complex interactions using a hierarchical, systems-based approach that integrates regional-to-continental spatial scales across time and emerged as a field of study partially as a response to the need to understand and predict invasion (Heffernan et al. 2014, Dodds et al. 2021). A macrosystems approach provides a way to address the well-known problems that arise when using small-scale, short-term case studies at a handful of sites to understand and predict macroscale, long-term phenomena such as invasion (LaRue et al. 2021). Macrosystems approaches highlight the importance of complicated processes, interactions, and effects, including macroscale feedbacks (i.e., positive or negative feedback loops between macroscale factors), cross-scale interactions (i.e., mechanisms that interact across local scales through macroscales), and cross-scale emergences (i.e., local processes that accumulate or interact to produce macroscale processes; Heffernan et al. 2014). Macrosystems research also examines teleconnections (i.e., phenomena that connect distant geographic regions by movement of biotic or abiotic matter, energy, or information; Heffernan et al. 2014), such as the movements of garlic mustard from its native range to its introduced range. In figure 3, we highlight the most important variables for garlic mustard success and show how some of these key mechanisms (figure 2) can interact with each other to influence garlic mustard invasion at the macroscale, demonstrating macroscale feedbacks, cross-scale interactions, and cross-scale emergence.

By extending our conceptual model using the macrosystems approach (figure 3), we can see that some of the seemingly less important local variables in determining invasion success, such as soil abiotic conditions such as high soil nitrogen or phosphorus, may actually drive longer-term and initially lower density patterns of garlic mustard through interactions with earthworms or intraspecific competition (figures 2 and 3). Other indirect impacts such as the release of secondary compounds, which causes mycorrhizal fungi decline, leading to less growth in native competitors, have been extensively studied (Stinson et al. 2006, Wolfe et al. 2008) and do not appear to be large-scale drivers for invasion success. However, this is complicated by the intraspecific competition dynamics that have direct evolutionary effects on the production of allelochemicals in garlic mustard (Evans et al. 2016). Although not shown in figure 3, dispersal has the potential to change synergies at multiple scales by speeding up interactions at the local scale and disrupting the synchronization of garlic mustard with other species, thereby strengthening or weakening interactions across the landscape.

Taken together, these models (specifically the mechanisms identified as most variable across different contexts in figure 2 and the interactions in figure 3) elucidate important missing areas of research for garlic mustard. In designing future studies, it is important to be aware that confounding factors, issues with statistical inference, or methodological differences among studies can lead to misidentification of context dependency (Catford et al. 2022). As such, there is a need for standardized conceptual understanding and a quantitative definition of the size and delineation of a plot, site, and region in invasive plant studies to allow for comparison among studies and an improved understanding of scaling effects on garlic mustard invasion and impact. Future researchers should sample and compare invaded sites with measurements nested across spatial scales to identify landscape-scale and long-term invasion mechanisms, as well as the localized synergies among environmental variables that facilitate invasion. A macrosystems framework also can be used to explore garlic mustard's responses to long-term (i.e., “slow”) macroscale processes (Heffernan et al. 2014) such as climate change or land-use change and how these macroscale processes interact with local-scale processes such as soil conditions that act along the continuum of temporal scales (figure 2).

Since 2008, investigators have begun incorporating aspects of a macrosystems approach, either explicitly or implicitly, in their exploration of plant invasion ecology (e.g., Fraterrigo et al. 2014, Cabra-Rivas et al. 2016, Iannone et al. 2016, Guo et al. 2017, Nunez-Mir et al. 2017), including garlic mustard invasion (e.g., Van Riper et al. 2010, Waller et al. 2016, Clark et al. 2018, Huebner et al. 2018, Urbanowicz et al. 2019). Some of these garlic mustard studies have found variable or contradictory results when analyzing factors at different spatial scales, which may be an indication of cross-scale interactions that are not accounted for (Dixon Hamil et al. 2016, LaRue et al. 2021) and emphasizes the importance of ecological context in understanding garlic mustard invasion. For example, Van Riper and colleagues (2010) found no relationship between light availability and garlic mustard success at the plot level (0.5 square meters), but a significant negative relationship between garlic mustard seedling cover and light availability at the larger site level (at least 0.15 hectares). At a larger landscape scale, forest edge occurrence of garlic mustard was negatively associated with elevation in each of two ecoregions in Massachusetts but positively associated with elevation when the two ecoregions were considered together (Urbanowicz et al. 2019). These studies highlight the promise of a macrosystems approach to understanding and forecasting garlic mustard invasion.

## Conclusions

Research on garlic mustard, and many other invasive species, has begun to converge on the understanding that invasion success is dependent on ecological context. A thorough investigation of how ecological context predicts plant invasion success will require more than an accumulation of short-term, small-scale studies. Instead, multisite, multiscale, longitudinal approaches, driven by a coordinated set of hypotheses and using consistent methods across a large geographic scale, will be most effective. This coordinated approach is appropriate for both emerging and established collaborative research networks, such as the Ecological Research as Education Network or the National Ecological Observatory Network (e.g., Dodds et al. 2021) that may include community scientists (e.g., Crall et al. 2015). The conceptual models proposed in the present article can guide hypothesis development and design of future multisite, long-term macroscale studies of garlic mustard, and may also be useful for other invasive plants. By designing multisite studies that consider how population traits vary over temporal and spatial scales (figure 2), while also considering key interactions within and across scales (figure 3), we will refine our understanding of garlic mustard invasion—specifically, which mechanisms are generalizable across sites versus those that are site specific. This approach to studying invasion can also inform research of other widespread invaders of consequence.

## Supplementary Material

biac012_Supplemental_Files

## Notes

Vikki L. Rodgers (vrodgers@babson.edu) is a professor of ecology and chair of the Math and Science Division at Babson College, in Babson Park, Massachusetts, in the United States. Sara E. Scanga (sescanga@utica.edu) is a professor and chair of biology in the Department of Biology at Utica University, in Utica, New York, in the United States. Rodgers and Scanga are co-first authors of this article. Mary Beth Kolozsvary is an associate professor of environmental studies and sciences at Siena College, in Loudonville, New York, in the United States. Danielle E. Garneau is an associate professor of environmental science in the Center for Earth and Environmental Science at State University of New York Plattsburgh, in Plattsburgh, New York, in the United States. Jason S. Kilgore is an associate professor of biology and coordinator for the Environmental Science Major at Washington and Jefferson College, in Washington, Pennsylvania, in the United States. Laurel J. Anderson is the Morris Family Professor of Natural Sciences at Ohio Wesleyan University, in Delaware, Ohio, in the United States. Kristine N. Hopfensperger is a professor of biology and is the director of the Environmental Science Program at Northern Kentucky University, in Highland Heights, Kentucky, in the United States. Anna G. Aguilera is an associate professor of biology at Simmons University, in Boston, Massachusetts, in the United States. Rebecca A. Urban is a professor of biology and is the program director of the Environmental Science Program at Lebanon Valley College, in Annville, Pennsylvania, in the United States. Kevyn J. Juneau is an assistant professor of conservation and environmental science at the University of Wisconsin–River Falls, in River Falls, Wisconsin, in the United States.
