# Supplementary material for: Where Is Garlic Mustard? Understanding the Ecological Context for Invasions of Alliaria petiolata
Source: Bioscience. 2022 Mar 30;72(6):521–37. doi: 10.1093/biosci/biac012 (PMC9169898; doi:10.1093/biosci/biac012)
Supplement: biac012_Supplemental_Files [file biac012_supplemental_files.zip › Rodgers_et-al_Author_Contributions_Supplementary_File.docx]

**Author contributions**

VLR, SES, JSK, LJA, and KNH conceived the article topic and design. SES, MBK, and DEG handled analysis and interpretation of data, as well as producing figure 1. VLR produced figures 2 and 3. MBK managed the references for the group. All authors participated in drafting the article. VLR, SES, MBK, DEG, JSK, LJA, KNH, AGA, and RAU participated in reviewing and revising the article for content.

Authors:

Vikki L. Rodgers (VLR)

Sara E. Scanga (SES)

Mary Beth Kolozsvary (MBK)

Danielle E. Garneau (DEG)

Jason S. Kilgore (JSK)

Laurel J. Anderson (LJA)

Kristine N. Hopfensperger (KNH)

Anna G. Aguilera (AGA)

Rebecca A. Urban (RAU)

Kevyn J. Juneau (KJJ)
